# Functional retrogression of LOFSEPs in specifying floral organs in barley

**DOI:** 10.1007/s42994-024-00182-4

**Published:** 2024-10-15

**Authors:** Chaoqun Shen, Xiujuan Yang, Duoxiang Wang, Gang Li, Matthew R. Tucker

**Affiliations:** 1 School of Agriculture, Food and Wine, Waite Research Institute, The University of Adelaide, Urrbrae, SA 5064 Australia; 2 Department of Plant Pathology, College of Plant Protection, Nanjing Agricultural University, Nanjing, 210095 China

**Keywords:** Barley, LOFSEP, MADS6, Floral organs

## Abstract

The barley genome encodes a complete set of MADS-box proteins sharing homology with components of the ABCDE model, which explains the molecular basis of floral organ identity in angiosperm flowers. Although the E-class members are universally expressed across floral whorls and crucial for flower development in Arabidopsis and rice, the functional role of the barley E-class LOFSEP subfamily (comprising MADS1, MADS5, and MADS34) remains elusive, particularly during spikelet formation. Here, we characterize the single, double and triple *lofsep* mutants in barley in an attempt to overcome the anticipated genetic redundancy. Surprisingly, loss of function of all LOFSEP members only disturbs lemma development, either converting this hull organ into a leaf-like structure or reducing its size. The inner organs, including lodicules, anthers and pistil remain unaffected. A systematic interrogation of how ABCDE class genes are affected in all whorls of the mutants was undertaken. Generally, in the lemma and palea of the *lofsep* mutants, A- and E-class genes are hyperactivated, B- and C- classes are slightly repressed, and D-class genes show unchanged expression in these inner organs. Intriguingly, loss of function of *MADS6*, an *AGL6* member closely related to the E-class genes, leads to most organs being transformed into lemma-like organs with new spikelets generated from the center of the flower. Contrasting with rice, these findings suggest barley LOFSEPs may have regressed in determining floral organ identity, and this could be partially compensated by HvMADS6.

**Supplementary Information:**

The online version contains supplementary material available at 10.1007/s42994-024-00182-4.

## Introduction

In the angiosperms, flower morphology exhibits remarkable diversity (Moyroud and Glover 2017). In model plants, such as Arabidopsis, the flower shows a typical whorl-based anatomy common to eudicots, with each whorl comprising specific organs: sepals in whorl 1, petals in whorl 2, stamens in whorl 3, and carpels in whorl 4 (Alvarez-Buylla et al. 2010; Brightbill and Sung 2022). In economically important cereal monocots, such as rice, the flower, known as the spikelet, follows a similar whorl pattern: lemma and palea in whorl 1, lodicules (equivalent to petals) in whorl 2, stamens in whorl 3, and the carpel in whorl 4 (Yoshida and Nagato 2011). Similarly, the barley spikelet is composed of 4 whorls in the same pattern as the rice spikelet, except for two major differences. The barley lemma develops an awn, which is a needle-like structure that extends from the tip of lemma. The awn is believed to deter seed predators and enhance sink strength for grain production (Sanchez-Bragado et al. 2023; Zhang et al. 2024). Instead of six stamens, each barley spikelet has three stamens in its third whorl, a common trait in the grass family (Cocucci and Anton 1988).

The ABCDE model explains the molecular basis for floral organ specification in each whorl (Causier et al. 2010). The five classes of genes encode homeotic proteins, named A-, B-, C-, D- and E-class, and govern the development of floral organs in a spatiotemporal manner. Almost every homeotic gene in the ABCDE model encodes a MADS-box protein, except for the A-class gene, *APETALA2* (*AP2*) (Bowman et al. 1989, 1991; Coen and Meyerowitz 1991; Jofuku et al. 1994; Theissen 2001; Weigel and Meyerowitz 1994). In Arabidopsis, A-class genes specify the sepals in whorl 1, A- and B-classes specify petals in whorl 2, B- and C-class genes specify stamens in whorl 3, and the single C-class gene (*AGAMOUS*) specifies the carpel in whorl 4. Furthermore, D-class genes control ovule formation within the carpel, and E-class members function as ‘glue’ proteins in every whorl to complex with the A-, B-, C- and D-class proteins (Bowman and Moyroud 2024). Apart from the core members, other MADS-box genes are also involved in floral organ patterning. For example, *AGAMOUS LIKE 6* (*AGL6*)-clade genes are closely related to E-class genes and are expressed in all whorls (Dreni and Zhang 2016).

The diversification of MADS-box genes among species contributes to the vast diversity of flower patterns in the angiosperms (Becker and Theißen 2003). While much of the early work in this field was completed in Arabidopsis and Antirrhinum, a well-structured ABCDE model has now been completed in rice, thanks primarily to phylogenetic comparison, analysis of mutants and RNAi lines, and studies on molecular function (Dreni 2023). A-class genes include *MADS14* and *MADS15*, required for lemma and palea formation (Wu et al. 2017). The B-class genes are *MADS2*, *MADS4* and *MADS16*, which specify lodicules and stamens (Nagasawa et al. 2003; Yao et al. 2008). The C-class genes, *MADS3* and *MADS58*, are expressed in stamens and carpels and develop the inner organs with gametophytes (Yamaguchi et al. 2006). D-class genes include *MADS13* and *MADS21*, and the former is required for ovule initiation (Dreni et al. 2007). The E-class genes are divided into two clades, LOFSEP including *MADS1*, *MADS5* and *MADS34*, and SEP3-like including *MADS7* and *MADS8* (Kuijer et al. 2021; Zahn et al. 2005). *MADS6* is the functional AGL6-class member in rice, interacting with other classes (Li et al. 2011). Among all MADS-box families, the E-class genes are of particular interest because they are predicted to be involved in development of all whorls in the spikelet. Moreover, the role of LOFSEP members such as *OsMADS5* and *OsMADS34* (and *HvMADS1* in barley), have additional roles in the regulation of inflorescence architecture in rice and barley, respectively (Gao et al. 2010; Li et al. 2021; Zhu et al. 2022).

Like rice, the barley genome encodes a complete set of ABCDE genes, however the exact functions of most remain unknown (Kuijer et al. 2021; Mascher et al. 2021). Previous transcriptional profiling of barley MADS-box genes revealed that the E-class members, and the closely related AGL6-family gene *HvMADS6*, are expressed in the young inflorescence and spikelet, predominantly in the lemma and palea (Kuijer et al. 2021; Liu et al. 2020), indicating a potential role in regulating floral organ patterning. Due to rapid developments in CRISPR/Cas9 mediated genome editing (Ma et al. 2015), their function can now be specifically addressed. Previously, we reported the role of two barley E-class MADS-box genes, highlighting their temperature-dependent molecular function. *HvMADS1*, from the LOFSEP clade, maintains spike architecture in barley under high temperature by controlling cytokinin signalling, regulates awn and lemma growth by promoting cell proliferation, and interacts with A-class genes to activate downstream targets (Li et al. 2021; Zhang et al. 2024). *HvMADS8*, from the SEP3 clade, restricts carpel cell activity and promotes ovule initiation under high temperature conditions (Shen et al. 2024). Remarkably, neither *hvmads1* nor *hvmads8* display altered floral organ development at normal ambient temperatures, suggestive of gene redundancy. The loss of all LOFSEPs in rice converts all whorls of floral organs to leaf-like structures (Wu et al. 2018), but whether the barley LOFSEPs have a similar role in floral organ identity remains unclear.

Here, we engineered and studied all single, double, and triple mutants for the barley LOFSEP clade. Surprisingly, even though barley LOFSEPs are expressed in all floral organs, the *Hvmads1/5/34* triple mutant only exhibited alterations to lemma identity. In contrast, knockout mutants of *HvMADS6* exhibited severe changes in floral organ patterning, leading to the transformation of palea, lodicules, and stamens into lemma-like organs and the formation of new spikelets. These findings provide evidence for functional retrogression of LOFSEPs in specifying floral organs in barley, with *MADS6* appearing as a potential substitute for this role compared to rice. Thus, diverged evolutionary strategies involving MADS-box genes appear to have been recruited in two major modern crops.

## Results

### LOFSEP members redundantly regulate lemma identity in barley

**Fig. 1:**
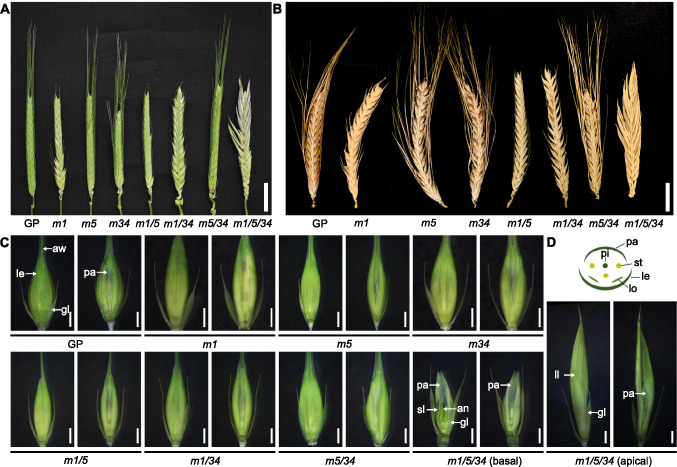
Phenotypes of *lofsep* mutants in barley. Young (**A**) and mature (**B**) inflorescence phenotypes of wild-type plants and *lofsep* mutants in GP background. Scale bars, 2 cm. **C** Spikelets of wild-type and *lofsep* mutants at W9.0. Scale bars, 2 mm. **D** Diagrammatic representation of wild-type floral structure. GP, Golden Promise. *m1*, *Hvmads1* single mutant. *m5*, *Hvmads5* single mutant. *m34*, *Hvmads34* single mutant. *m1/5*, *Hvmads1 Hvmads5* double mutant. *m1/34*, *Hvmads1 Hvmads34* double mutant. *m5/34*, *Hvmads5 Hvmads34* double mutant. *m1/5/34*, *Hvmads1 Hvmads5 Hvmads34* triple mutant. le, lemma; pa, palea; st, stamen; gl, glume; sl, short lemma-like structure; ll, leaf-like lemma structure; aw, awn

To investigate the role of LOFSEP members in barley, we employed the CRISPR/Cas9 system in the Golden Promise (GP) cultivar, generating single, double, and triple mutants of three *LOFSEP* MADS-box genes: *HvMADS1*, *HvMADS5*, *HvMADS34*. Due to the target sites being located on the first exon of the coding sequence, all induced insertions or deletions resulted in frameshift mutations, and each mutant is unable to encode a complete MADS-box protein (Li et al. 2021; Fig. S1). A previous study had shown that none of the single or double mutants showed visible abnormalities in inflorescence architecture (Fig. 1A and B) (Li et al. 2021). Likewise, the *Hvmads1/5/34* triple mutants also maintained an inflorescence architecture resembling that of wild-type (Fig. 1A and B).

**Fig. 2:**
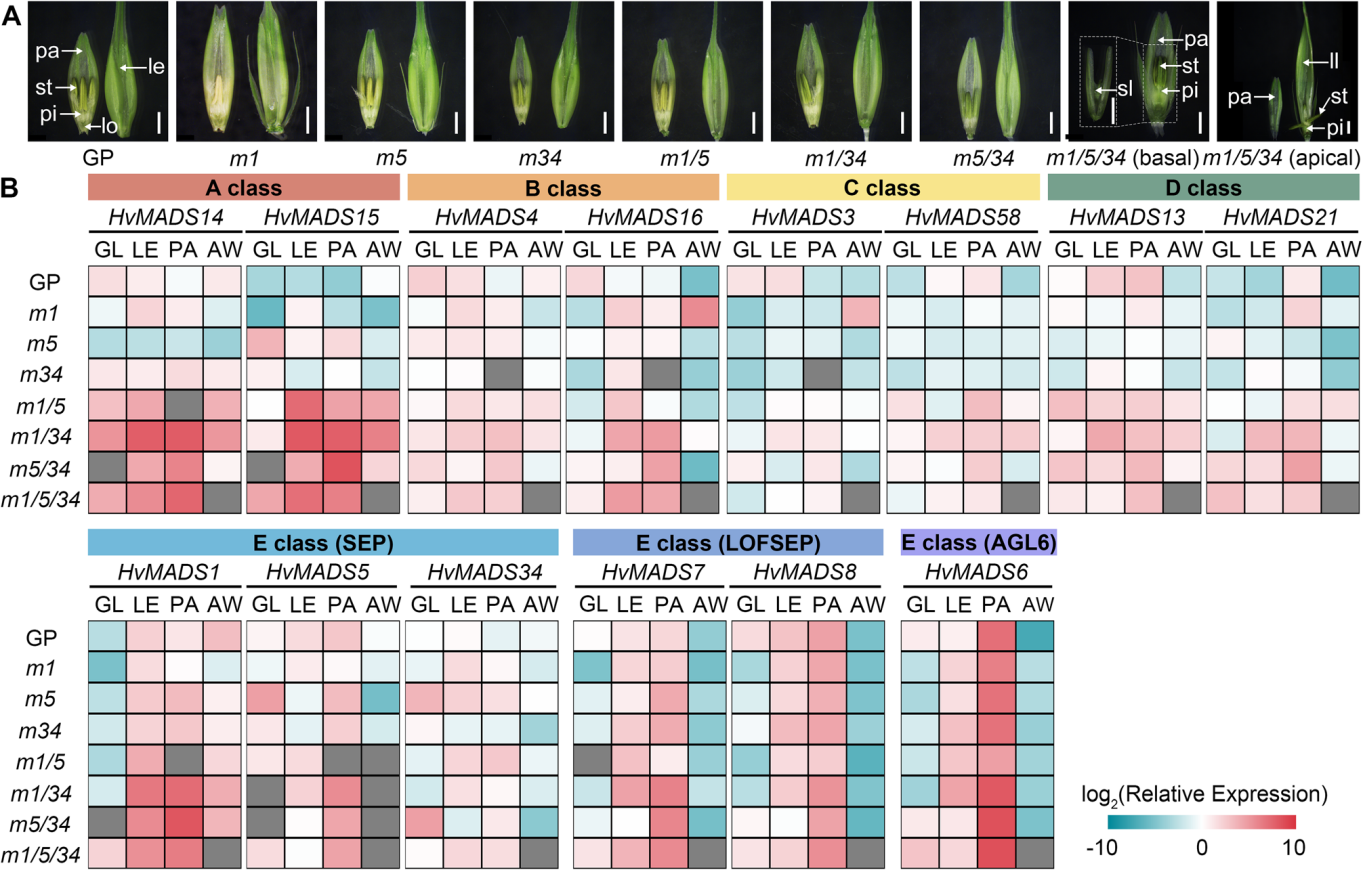
*LOFSEP* genes control the development of floral organs in outer whorls. **A** Lemma and palea with inner floral organs of wild-type and *lofsep* mutants at W9.0. Insets indicate high magnification of palea. Scale bars, 2 mm. le, lemma; pa, palea; lo, lodicule; st, stamen; pi, pistil; sl, short lemma-like structure; ll, leaf-like lemma structure. **B** Heatmap representation of the expression of A-class, E-class, and AGL6 members in outer floral organs. The heatmap was generated using the log_2_ (relative expression value) in wild-type and *lofsep* mutants. Red represents higher relative expression level compared with the internal control (*HvActin7*), and blue represents lower relative expression level. Gray boxes indicate samples where gene expression was not detected. AW, awn. LE, lemma. PA, palea. GL, glumes. GP, Golden Promise. *m1*, *Hvmads1* single mutant. *m5*, *Hvmads5* single mutant. *m34*, *Hvmads34* single mutant. *m1/5*, *Hvmads1 Hvmads5* double mutant. *m1/34*, *Hvmads1 Hvmads34* double mutant. *m5/34*, *Hvmads5 Hvmads34* double mutant. *m1/5/34*, *Hvmads1 Hvmads5 Hvmads34* triple mutant

Florets in wild-type barley consist of five concentric whorls, including two glumes, a lemma, and a palea as the first and second outer whorls, and two lodicules, three stamens, and a pistil as three inner floral whorls (Fig. 1D). Compared to the wild-type florets, the *Hvmads1*, *Hvmads1*/*5*, *Hvmads1*/*34* and *Hvmads1*/*5*/*34* florets exhibited shorter awns (Figs. 1A and B, S2), while the remaining floral organs maintained their wild-type structure (Figs. 1C and 2A). It is worth noting that the spikelets at the basal part the spike of *Hvmads1*/*5*/*34* triple mutants displayed different defects in the lemma, characterized by significant shortening and incomplete development of the awn and central lemma structure. Conversely, in the spikelets at the apical part of the spike, the lemma transformed into a significantly elongated leaf-like structure (Fig. 1C and 2A). As a result of the reduced awn length, which was reported to contribute to spike photosynthesis of the grain (Zhang et al. 2024), and the defect in the lemma structure, the seed setting rates were reduced in all the *lofsep* mutants (Fig. S3). In all *Hvmads1*-related mutants, the grain width was reduced whereas the grain length was increased in the *Hvmads1*/*5*, *Hvmads1*/*34* and *Hvmads1*/*5*/*34* mutants (Fig. S4), likely a consequence of leaf-like structures observed in these mutants. These findings collectively revealed the predominant role of HvMADS1 in regulating awn length, alongside a highly redundant function among the three *LOFSEP* members in regulating lemma identity.

### *LOFSEP* members negatively regulate expression of floral homeotic genes

**Fig. 3:**
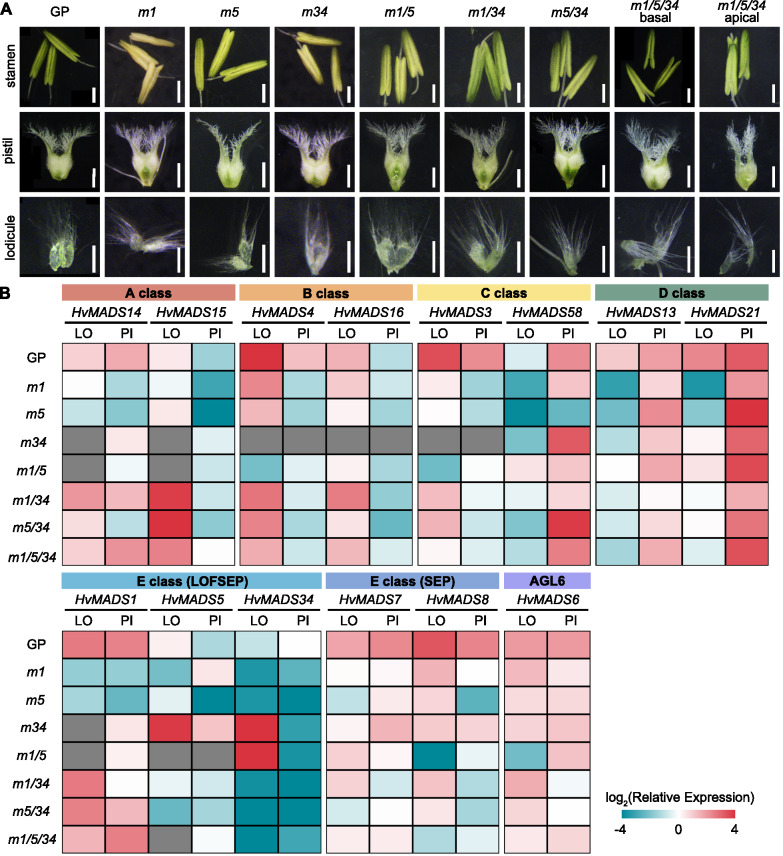
*LOFSEP* genes control the development of floral organs in inner whorls. **A** Stamen, pistil and lodicule of wild-type and *lofsep* mutants at W9.0. Scale bars, 1 mm. **B** Heatmap representation of the expression of A-, B-, C-, D-, E-class, and AGL6 members in inner floral organs. The heatmap was generated using the log_2_ (relative expression value) in wild-type and *lofsep* mutants. Red represents higher relative expression level compared with the internal control (*HvActin7*), and blue represents lower relative expression level. Gray boxes indicate samples where gene expression was not detected. LO, lodicule. PI, pistil. GP, Golden Promise. *m1*, *Hvmads1* single mutant. *m5*, *Hvmads5* single mutant. *m34*, *Hvmads34* single mutant. *m1/5*, *Hvmads1 Hvmads5* double mutant. *m1/34*, *Hvmads1 Hvmads34* double mutant. *m5/34*, *Hvmads5 Hvmads34* double mutant. *m1/5/34*, *Hvmads1 Hvmads5 Hvmads34* triple mutant

To further study the regulatory function of HvMADS1, HvMADS5, and HvMADS34, qRT-PCR analysis was performed to examine the transcript levels of the other floral homeotic genes. Young inflorescences at stage W3.5 (floret primordium) and W5.0 (primordium of all floral organ present) as well as floral organs at stage W9.5 were examined in the *lofsep* mutants and the wild-type (Figs. 2, 3 and S5A).

In young inflorescences, the transcripts of two *AP1/FUL*-like A-class genes, *HvMADS14* and *HvMADS15*, were significantly elevated in all *lofsep* mutants, indicating a negative regulation of *LOFSEP* genes on A-class members (Fig. S5B). B-class members, the *PI*-like gene *HvMADS4* and the *AP3*-like gene *HvMADS16*, showed decreased expression levels at W3.5, but were subsequently upregulated at W5.0 (Fig. S5B). The expression levels of *AG* lineage C-class genes *HvMADS3* and *HvMADS58*, along with the two D-class genes *HvMADS13* and *HvMADS21*, showed comparable expression patterns in *lofsep* mutants and wild-type, except for a slight upregulation of *HvMADS3* at W5.0 (Fig. S5B). Among E-class genes, *HvMADS1* exhibited significant upregulation in *HvMADS5* and *HvMADS34*-related mutants. Additionally, the two *SEP3* genes, *HvMADS7* and *HvMADS8*, and the *AGL6*-like gene *HvMADS6*, showed moderately increased expression levels at W5.0 (Fig. S5B).

In the outer floral organs of *lofsep* mutants, the transcripts of *HvMADS14* and *HvMADS15* showed a dramatic increase, especially in the lemma and palea of double and triple mutants (Fig. 2B). Similarly, in line with the expression detected in young inflorescences at W5.0, the expression levels of *HvMADS6* and *HvMADS7* were slightly upregulated in the lemma and palea of double and triple mutants (Fig. 2B). Meanwhile, we also analysed the expression of the *LOFSEP* genes in their respective mutant backgrounds. *HvMADS1* expression was found to be upregulated in the *Hvmads1/34*, *Hvmads5/34*, and *Hvmads1/5/34* background, indicating potential feedback regulation within the *LOFSEP* family (Fig. 2B). The expression level of *HvMADS5* decreased to varying degrees in the mutants, while *HvMADS34* showed a slight upregulation in glumes, palea and lemma in *lofsep* mutants (Fig. 2B).

Taken together, these findings imply that the barley LOFSEP members function redundantly as upstream inhibitors of floral homeotic genes, especially the A-class genes. Within the barley LOFSEP family, *HvMADS1* may also be regulated by the three genes, including itself, suggesting a regulatory feedback loop that fine-tunes the expression of *HvMADS1*.

### Functional retrogression of barley LOFSEP members in inner floral organ regulation

As previously demonstrated in rice, the LOFSEP genes redundantly control floral meristem determinacy and the identities of floral organs. Notably, loss-of-function mutants show homeotic transformation of inner floral organs into leaf-like sterile lemmas, lemmas and paleas (Wu et al. 2018). To address whether *LOFSEP* genes have conserved functions in barley, we examined the morphology of inner floral organs in the *lofsep* mutants. Surprisingly, no changes in the number or morphology of the lodicule, stamens, and pistil were observed in any of the barley *lofsep* mutants (Fig. 3A).

We conducted a further analysis of the expression profile of all the floral homeotic genes in inner floral organs across all *lofsep* mutant backgrounds. This investigation aimed to uncover whether the functional divergence between barley and rice might stem from different regulatory roles between LOFSEP and other floral homeotic genes. In contrast to what was observed in rice, where the expression of B-class genes, C-class genes, SEP3-like E-class genes, and AGL6-like genes significantly decreased in *osmads1*, *lofsep* double, and triple mutants (Wu et al. 2018), our analysis in barley showed that the expression levels of most floral homeotic genes maintained similar patterns in *lofsep* mutants compared to wild-type in inner floral organs (Fig. 3B). The exceptions were a moderate increase in *HvMADS15* expression in double and triple mutant lodicules and a decrease in the expression of the B-class gene *HvMADS4* and C-class gene *HvMADS3* in *lofsep* mutants (Fig. 3B).

Within the LOFSEP members, we observed a decrease in the expression of *HvMADS1* in single and *Hvmads1/5* double mutants, while an increased expression level of *HvMADS5* was detected in *Hvmads34*. Additionally, *HvMADS34* was shown to be upregulated in the *Hvmads34* and the *Hvmads1/5*. These results further support the presence of feedback regulation among these *LOFSEP* members (Fig. 3B).

Collectively, the analysis of inner floral organs indicated a functional regression of *LOFSEP* members in regulating inner organ development, which may be partly attributed to the distinct regulation within barley *LOFSEP* and floral homeotic genes compared to rice.

### HvMADS6 is a key regulator of barley floral organ identity and meristem determinacy

The *AGL6*-like gene in rice was demonstrated to have *SEP*-like functions, as evidenced by the *osmads6-1* mutant exhibiting homeotic transformations in both outer and inner floral organs, including palea, lodicules and stamens. Furthermore, defective carpels and ovules and indeterminate meristems were also observed in *osmads6-1*, highlighting the roles of *OsMADS6* in regulating floral identity and floral meristem determinacy (Li et al. 2010). Therefore, we obtained the *Hvmads6* single mutant to investigate the biological role of *HvMADS6* in barley.

**Fig. 4:**
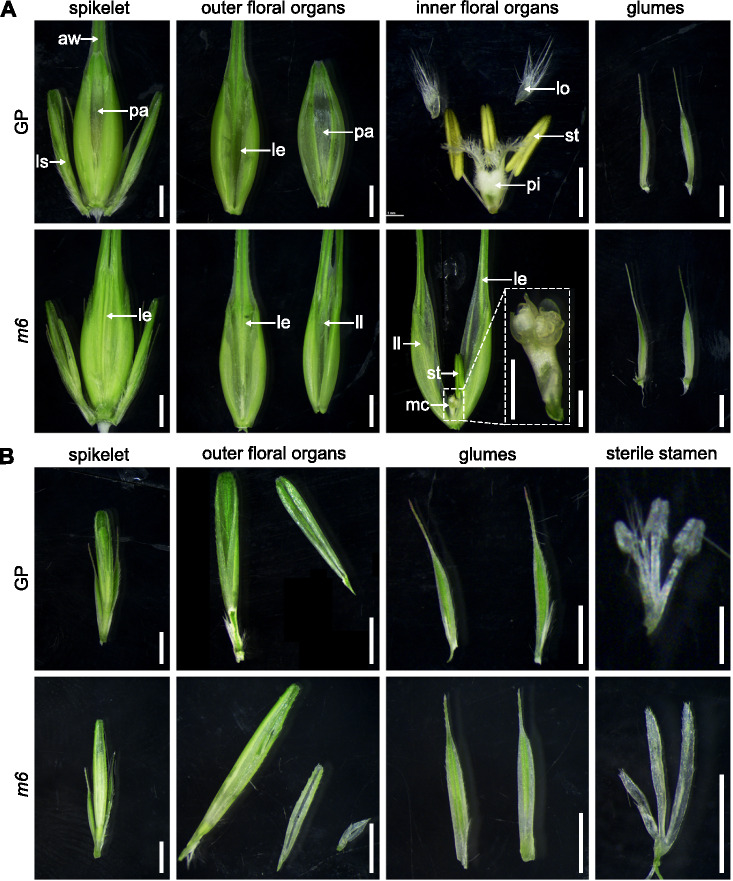
HvMADS6 mediates in the development of barley floral organs. **A** Phenotype of central spikelet. Scale bars, 2 mm. Insets indicate high magnification of mutated pistil. Scale bar, 1 mm. le, lemma; pa, palea; lo, lodicule; st, stamen; pi, pistil; mc, mosaic carpel; ll, lemma-like structure; aw, awn; ls, lateral spikelet. **B** Phenotype of the lateral spikelet. Scale bars, 2 mm. GP, Golden Promise. *m6*, *Hvmads6*

In the outer floral organs, the palea of the *Hvmads6* mutants transformed into a lemma-like structure, similar to that reported in rice. This reveals a conserved role of *HvMADS6* in specifying palea identity (Fig. 4A). Regarding the inner floral organs, the lodicules were entirely absent in *Hvmads6*, and the number of stamens decreased from three to one compared to wild-type, with ectopic lemma-like structures containing awns observed (Fig. 4A). Furthermore, the carpel in *Hvmads6* exhibited defects, being replaced by a mosaic carpel and a structure resembling a floral meristem at the centre of the flower (Fig. 4A). As a result, *Hvmads6* mutants were completely sterile and were unable to produce seed. These phenotypes observed in *Hvmads6* indicate the essential role of HvMADS6 in specifying floral organs and a potential role in regulating floral meristem determinacy in barley.

In addition to the abnormal phenotypes observed in the central spikelet, *Hvmads6* also showed defects in the lateral spikelets. In wild-type lateral spikelets, three degenerated sterile stamens are typically visible in the centre of the flower. However, in *Hvmads6*, these degenerated stamens are absent, replaced by three bract-like structures (Fig. 4B).

In summary, the disrupted development of floral organs in *Hvmads6* highlights a crucial role for HvMADS6 in determining organ identity, including the palea and all inner floral organs, as well as regulating floral meristem development. Based on these findings, we speculate that HvMADS6 function may partially compensate for the loss of *LOFSEP* genes responsible for inner floral organ development in barley.

## Discussion

Barley and rice are two representative species of cereal crops from the grass family. They share several common features, such as the prolific tillering architecture of the plant, a single headed inflorescence bearing multiple spikelets and small-sized starchy grains. Despite this situation, some significant differences are also notable. The rice inflorescence is a branched panicle, composed of primary and secondary branches bearing spikelets. The barley inflorescence is a non-branched spike, with spikelets directly attached to the inflorescence axis. As to the structure of the spikelet, lemma, palea, lodicules, stamens and pistil containing a single ovule, are arranged in a highly similar pattern in both barley and rice.

**Fig. 5:**
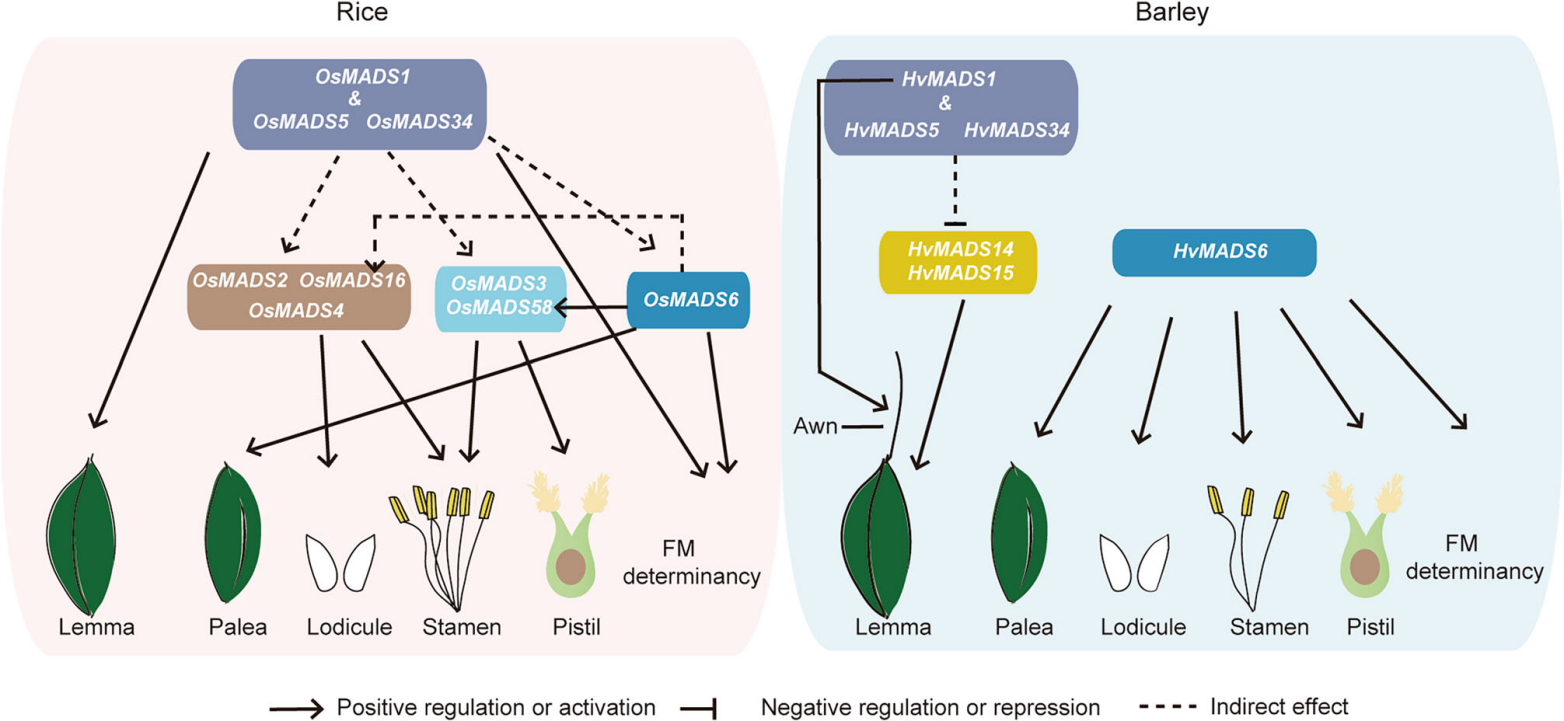
Working models of *LOFSEP*-mediated development of floral organs in rice and barley. Genetic interactions of MADS-box factors in regulating rice and barley floret organ morphogenesis. In rice, the *LOFSEP* genes were proposed to be upstream activators of B-class (*OsMADS2*, *OsMADS4* and *OsMADS16*), C-class (*OsMADS3* and *OsMADS58*) and *AGL6*-like (*OsMADS6*) genes to specify floral organs and FM determinacy. However, in barley, *LOFSEP* genes may act upstream of A-class genes (*HvMADS14* and *HvMADS15*) in directing lemma development, with *HvMADS6* acting as a substitute for key inner floral organ regulator

Utilizing the rice ABCDE model as a reference, close homologs of core MADS-box family members responsible for floral organ identification can be identified in the barley genome (Kuijer et al. 2021). In rice, the function of LOFSEPs from the E-class is to specify floral organs of all whorls and to constrain meristem activity after ovule formation (Fig. 5). Single mutant *osmads1*, *osmads34* and double mutants *osmads1 osmads5*, *osmads5 osmads34* gradually display exacerbated abnormalities in the lemma, stamens and especially in the pistil, which often exhibits an excessive capacity to generate new organs (Chen et al. 2006; Li et al. 2010; Wu et al. 2018). Remarkably, in the triple mutant *osmads1 osmads5 osmads34*, almost all whorls of floral organs become leaf-like structures, with 2–4 stamens remaining, and extra pistils and flowers being generated from the centre of spikelet (Wu et al. 2018). In striking contrast, single and double mutants of barley LOPSEPs maintain floral organ identities in all whorls, with only minor abnormalities observed, such as a shortened awn in mutants carrying *hvmads1*. Even in *hvmads1 hvmads5 hvmads34* triple mutants, only the identity of lemma is compromised, phenotypically ranging from a leaf-like structure to a retarded small organ, dependent on the spikelet position on the spike. As a result of the lemma identity change, the grain width was reduced whereas grain length increased in the *Hvmads1*-related double and triple mutants. This increase in grain length may improve the overall grain volume, as reported in the wheat SVP MADS-box gene mutant (Adamski et al. 2021). Despite some expression fluctuations of other ABCDE class genes, development and patterning of other floral organs are completely normal (Fig. 5).

AGL6 is closely related with E-class genes and acts as a ‘glue’ protein complexing with B-, C-, and D-class proteins in rice (Dreni and Zhang 2016). The rice *osmads6-1* mutant shows a phenotype in all whorls, including altered palea, mosaic stamens, extra glumes, an abnormal pistil and loss of meristem determinacy. By comparison, the barley *hvmads6* mutant displays more severe defects in floral organ identity. In most central spikelets of *hvmads6*, only one stamen remains, the pistil is converted to new spikelets located in the centre of the flower, and other organs are all transformed into lemma-like structures, indicated by their protruding awns. These phenotypes were also observed in the recently reported barley *mfo1* mutant, which carries loss-of-function mutations in *HvAGL6* (Sun et al. 2024). Even in the lateral spikelets of *hvmads6*, the retarded sterile stamens are converted to glume-like structures. These striking changes are reminiscent of the rice *lofsep* triple mutant and another double mutant, *osmads1-z osmads6-1*, which develop multiple glume-like organs instead of inner organs, and one additional spikelet-like organ in the flower center (Li et al. 2010; Wu et al. 2018). According to our phenotypic analysis, we speculate that the function of barley LOFSEPs in floral organ identification has regressed, whilst HvMADS6 may have supplanted LOFSEPs to play a more pivotal role in specifying organs and controlling meristem determinacy (Fig. 5). Consistent with this hypothesis, the wheat *TaAGL6* gene has been reported to be essential for maintaining proper expression of many floral genes, acting as a master regulator for all four whorls of floral organs and the spikelet meristem (Kong et al. 2022; Su et al. 2019).

Beyond floral organ patterning, LOFSEP genes also regulate inflorescence morphogenesis in both rice and barley, albeit through different molecular mechanisms. In rice panicles, *OsMADS5* and *OsMADS34* limit branching events to secondary branches, evidenced by *osmads5 osmads34* double mutants developing more secondary, and even tertiary and quaternary branches (Zhu et al. 2022). In barley spikes, none of the *lofsep* mutants display obvious phenotypes in inflorescence architecture under normal ambient temperatures. However, under high temperature conditions, the *hvmads1* mutant can be induced to develop a ‘branched’ inflorescence, via deregulation of downstream cytokinin signalling (Li et al. 2021). These findings again point towards functional diversity within the LOFSEP subclass between species, and more importantly, involvement in thermo-response. Elevated ambient temperatures often induce more active cell division and rapid growth before becoming heat stressed (Brown 1951; Ben-Haj-Salah and Tardieu 1995). These temperatures may challenge the morphological stability of crops domesticated from temperate climes, such as barley, compared to those that are adapted to warm tropical climates, such as rice. Compared to rice, barley LOFSEPs appear to have evolved to cope with environmental changes, a hypothesis that requires further investigation through future stress tests on additional LOFSEP genes in barley.

## Materials and methods

### Plant materials and growth conditions

The barley cultivar used in this study was Golden Promise. The monocot CRISPR/Cas9 system was used to obtain mutants (Ma et al. 2015). The target sites of *HvMADS1*, *HvMADS5*, *HvMADS34*, and *HvMADS6* were sequenced in Golden Promise and all showed 100% identity to the ‘Morex’ reference genome. The transgenic lines were created by *Agrobacterium*-mediated transformation (strain AGL1) as described previously (Harwood 2014). Independent T_0_ plants were genotyped using the Phire Plant Direct PCR Kit (Thermo Fisher Scientific), followed by Sanger sequencing of the amplified target sites (Australian Genome Research Facility, AGRF). For phenotypic observation and sample collection, barley plants were cultivated on cocopeat soil at controlled temperatures (15 °C day/10 °C night), with 50% humidity and a 16-h light/8-h dark photoperiod in growth chambers with a light intensity of ∼190 μmol m^−2^ s^−1^ (The Plant Accelerator, University of Adelaide).

### Morphological analysis

The barley inflorescences were photographed using a Nikon D5600 camera. The spikelets and floral organs at W9.0 from wild-type and mutants were photographed using a stereomicroscope equipped with a digital camera (Leica, MZ FLIII).

### RNA isolation and quantitative reverse transcription PCR analysis

Total RNA was isolated from young inflorescences (W3.5 and W5.0), glumes, lemma, palea, lodicule, stamen, and pistil using TRIzol reagent (Invitrogen) according to manufacturer’s instructions. cDNA synthesis and real-time quantitative reverse-transcription polymerase chain reaction (qRT-PCR) were performed according to Shen et al. (2024). The *HvActin7* gene was used as internal control. Three biological replicates were performed for each experiment. Data analysis was performed as previously described (Zhang et al. 2024). The relative expression level is the average of three biological replicates. The log_2_(relative expression value) is used for generating the heatmap using MeV software. All primers are listed in Supplemental Table 1.

### Accession numbers

The sequences for all barley genes used in this study are available in the Phytozome database (https://phytozome-next.jgi.doe.gov/). The accession numbers of genes mentioned are as follows: HvMADS14 (HORVU5Hr1G095630), HvMADS15 (HORVU2Hr1G063800), HvMADS4 (HORVU1Hr1G063620), HvMADS16 (HORVU7Hr1G091210), HvMADS3 (HORVU3Hr1G026650), HvMADS58 (HORVU1Hr1G029220), HvMADS13 (HORVU1Hr1G023620), HvMADS21 (HORVU1Hr1G064150), HvMADS1 (HORVU4Hr1G067680), HvMADS5 (HORVU7Hr1G025700), HvMADS34 (HORVU5Hr1G095710), HvMADS7 (HORVU7Hr1G054220), HvMADS8 (HORVU5Hr1G076400), HvMADS6 (HORVU6Hr1G066140).

## Supplementary Information

Below is the link to the electronic supplementary material.

Supplementary file1 (DOCX 24 kb)

Supplementary file2 (PDF 3609 kb)

Supplementary file3 (PDF 540 kb)

Supplementary file4 (PDF 841 kb)

Supplementary file5 (PDF 1794 kb)

Supplementary file6 (PDF 12051 kb)

Supplementary file7 (XLSX 13 kb)

## Data Availability

The authors declare that all data supporting the findings of this study are available within the article and its supplementary information files are available from the corresponding author upon request.

## Materials availability

This study did not generate new unique reagents. Plasmids and transgenic plant seeds generated in this study will be made available on request, but may require a completed Materials Transfer Agreement.
