# Supplementary material for: Functional retrogression of LOFSEPs in specifying floral organs in barley
Source: aBIOTECH. 2024 Oct 15;6(1):1–11. doi: 10.1007/s42994-024-00182-4 (PMC11889289; doi:10.1007/s42994-024-00182-4)
Supplement: Supplementary file 1 — Supplementary file1 (DOCX 24 kb) [file 42994_2024_182_MOESM1_ESM.docx]

**Functional Retrogression of LOFSEPs in Specifying Floral Organs in Barley**

Chaoqun Shen^1,3^, Xiujuan Yang^1,3^, Duoxiang Wang^1^, Gang Li^2,*^, Matthew R. Tucker^1,*^

^1^ Waite Research Institute, School of Agriculture, Food and Wine, The University of Adelaide, Urrbrae, South Australia, 5064, Australia

^2^ Department of Plant Pathology, College of Plant Protection, Nanjing Agricultural University, Nanjing 210095, China

^3^ These authors contributed equally

* Correspondence: [matthew**.**tucker@adelaide**.**edu**.**au](mailto:matthew.tucker@adelaide.edu.au) (M.R.T); [gang.li@njau.edu.cn](mailto:gang.li@njau.edu.cn) (G.L.)

**Supplementary Figure S1. Diagrams of the *Hvmads1/5/34* and *Hvmads6* mutated sites.**

Schematic representations of gene organization showing untranslated regions (grey boxes), exons (red boxes), and introns (red lines). The first exon was targeted by CRISPR-Cas9 to generate *Hvmads1/5/34* and *Hvmads6* mutants. Nucleotides in red indicate the target sites for mutation; blue nucleotides indicate the inserted sequence, and blue dashes indicate deletions.

**Supplementary Figure S2. Average awn length of *lofsep* mutants and wild-type plants.** Data shown as mean ± s.d. GP, Golden Promise. *m1*, *Hvmads1* single mutant. *m5*, *Hvmads5* single mutant. *m34*, *Hvmads34* single mutant. *m1/5*, *Hvmads1 Hvmads5* double mutant. *m1/34*, *Hvmads1 Hvmads34* double mutant. *m5/34*, *Hvmads5* *Hvmads34* double mutant. *m1/5/34*, *Hvmads1 Hvmads5 Hvmads34* triple mutant. Different letters indicate significant differences derived from one-way ANOVA with Tukey’s multiple comparisons test (*P* < 0.05).

**Supplementary Figure S3. Seed setting rate of *lofsep* and *Hvmads6* mutants and wild-type plants.** Data shown as mean ± s.d. GP, Golden Promise. *m1*, *Hvmads1* single mutant. *m5*, *Hvmads5* single mutant. *m34*, *Hvmads34* single mutant. *m1/5*, *Hvmads1 Hvmads5* double mutant. *m1/34*, *Hvmads1 Hvmads34* double mutant. *m5/34*, *Hvmads5* *Hvmads34* double mutant. *m1/5/34*, *Hvmads1 Hvmads5 Hvmads34* triple mutant, m6, *Hvmads6* single mutant. Different letters indicate significant differences derived from one-way ANOVA with Tukey’s multiple comparisons test (*P* < 0.05).

**Supplementary Figure S4. Grain size of *lofsep* mutants and wild-type plants.** Comparison of the grain width (**A**) and grain length (**B**) of all the *lofsep* mutants. Data shown as mean ± s.d. GP, Golden Promise. *m1*, *Hvmads1* single mutant. *m5*, *Hvmads5* single mutant. *m34*, *Hvmads34* single mutant. *m1/5*, *Hvmads1 Hvmads5* double mutant. *m1/34*, *Hvmads1 Hvmads34* double mutant. *m5/34*, *Hvmads5* *Hvmads34* double mutant. *m1/5/34*, *Hvmads1 Hvmads5 Hvmads34* triple mutant. Different letters indicate significant differences derived from one-way ANOVA with Tukey’s multiple comparisons test (*P* < 0.05).

**Supplementary Figure S5. Expression analysis of floral homeotic genes in the immature inflorescences at W3.5 and W5.0 of *lofsep* mutants and the wild-type backgrounds.**

**A** Morphology of wild-type inflorescence at stages W3.5 and W5.0. Scale bars, 1 mm. **B** Heatmap representation of the expression of A, B, C, D, E-class, and AGL6 members in inflorescences at stage W3.5 and W5.0. GP, Golden Promise. The heatmap was generated using the log_2_ (relative expression value) in wild type and *lofsep* mutants. Red represents higher relative expression level compared with the internal control (*HvActin7*), and blue represents lower relative expression level. Gray boxes indicate samples where gene expression was not detected. *m1*, *Hvmads1* single mutant. *m5*, *Hvmads5* single mutant. *m34*, *Hvmads34* single mutant. *m1/5*, *Hvmads1 Hvmads5* double mutant. *m1/34*, *Hvmads1 Hvmads34* double mutant. *m5/34*, *Hvmads5* *Hvmads34* double mutant. *m1/5/34*, *Hvmads1 Hvmads5 Hvmads34* triple mutant.
