# Supplementary material for: Functional retrogression of LOFSEPs in specifying floral organs in barley
Source: aBIOTECH. 2024 Oct 15;6(1):1–11. doi: 10.1007/s42994-024-00182-4 (PMC11889289; doi:10.1007/s42994-024-00182-4)
Supplement: Supplementary file 2 — Supplementary file2 (PDF 3609 kb) [file 42994_2024_182_MOESM2_ESM.pdf]

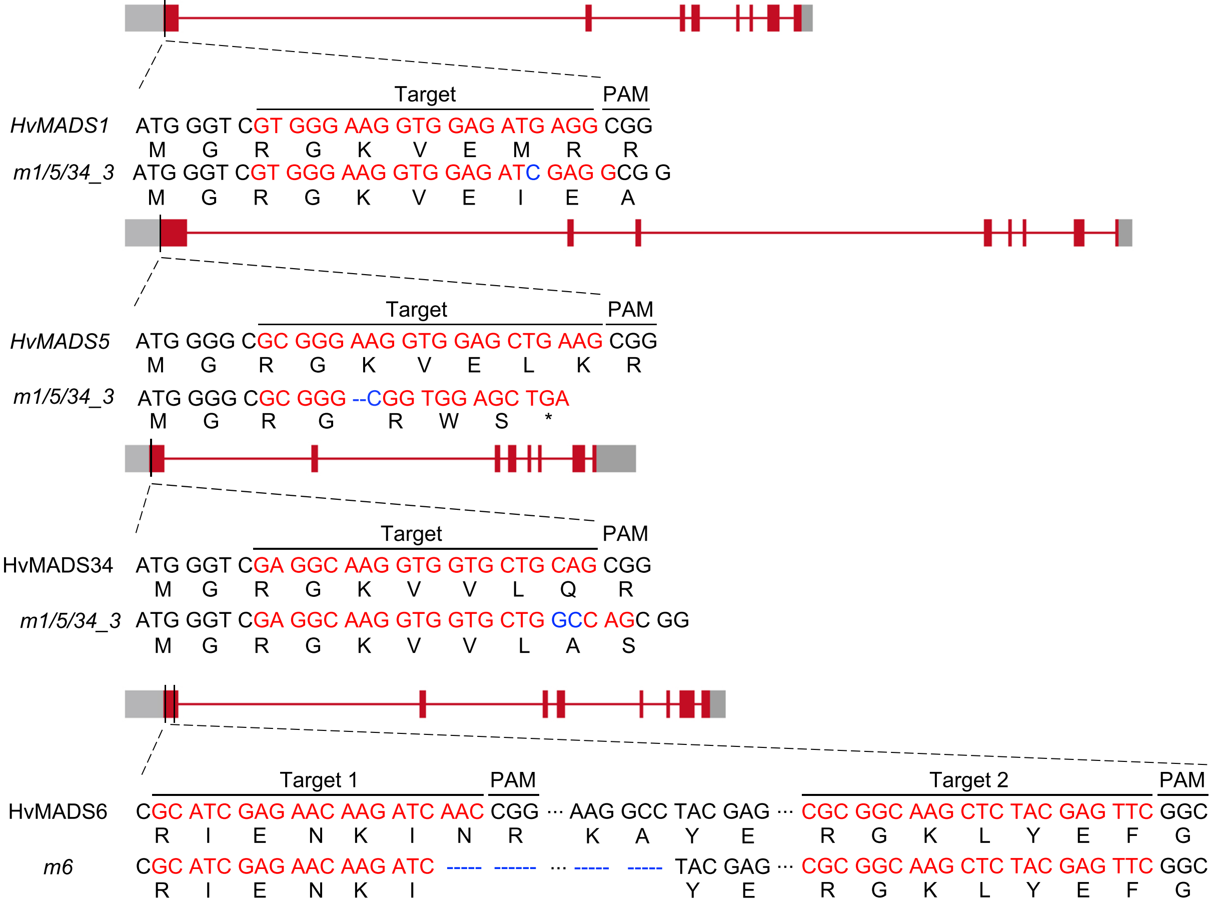

Target
PAM
HvMADS1 ATG GGT CGT GGG AAG GTG GAG ATG AGG CGG
M G R G K V E M R R
m1/5/34_3 ATG GGT CGT GGG AAG GTG GAG ATC GAG GCG G
M G R G K V E I E A
Target
PAM
HvMADS5 ATG GGG CGC GGG AAG GTG GAG CTG AAG CGG
M G R G K V E L K R
m1/5/34_3 ATG GGG CGC GGG --CGG TGG AGC TGA
M G R G R W S *
Target
PAM
HvMADS34 ATG GGT CGA GGC AAG GTG GTG CTG CAG CGG
M G R G K V V L Q R
m1/5/34_3 ATG GGT CGA GGC AAG GTG GTG CTG GCC AGC GG
M G R G K V V L A S
Target 1
PAM
Target 2
PAM
HvMADS6 CGC ATC GAG AAC AAG ATC AAC CGG ··· AAG GCC TAC GAG ··· CGC GGC AAG CTC TAC GAG TTC GGC
R I E N K I N R K A Y E R G K L Y E F G
m6 CGC ATC GAG AAC AAG ATC ----- ------ ··· ----- ----- TAC GAG ··· CGC GGC AAG CTC TAC GAG TTC GGC
R I E N K I Y E R G K L Y E F G
