# Supplementary figures and images for: Functional retrogression of LOFSEPs in specifying floral organs in barley
Source: aBIOTECH. 2024 Oct 15;6(1):1–11. doi: 10.1007/s42994-024-00182-4 (PMC11889289; doi:10.1007/s42994-024-00182-4)

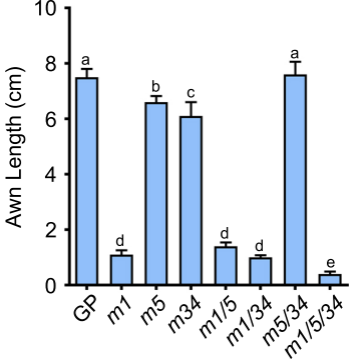

Supplement: Supplementary file 3 — Supplementary file3 (PDF 540 kb) [file 42994_2024_182_MOESM3_ESM.pdf]

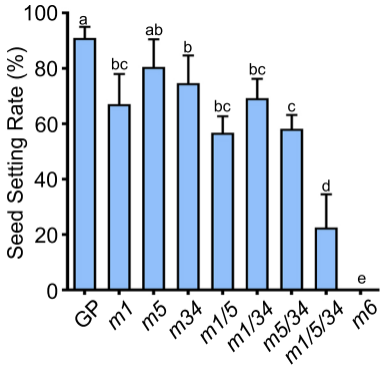

Supplement: Supplementary file 4 — Supplementary file4 (PDF 841 kb) [file 42994_2024_182_MOESM4_ESM.pdf]

**A**

Grain Width (cm)

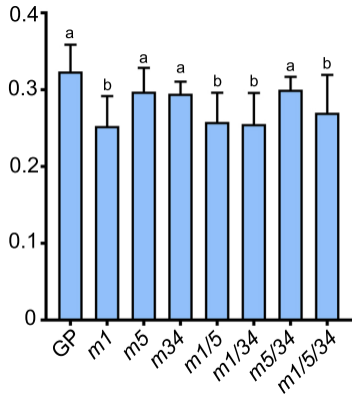**B**

Grain Length (cm)

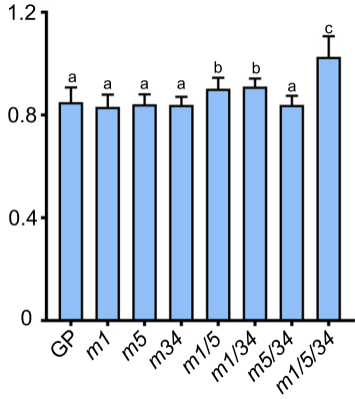

Supplement: Supplementary file 5 — Supplementary file5 (PDF 1794 kb) [file 42994_2024_182_MOESM5_ESM.pdf]

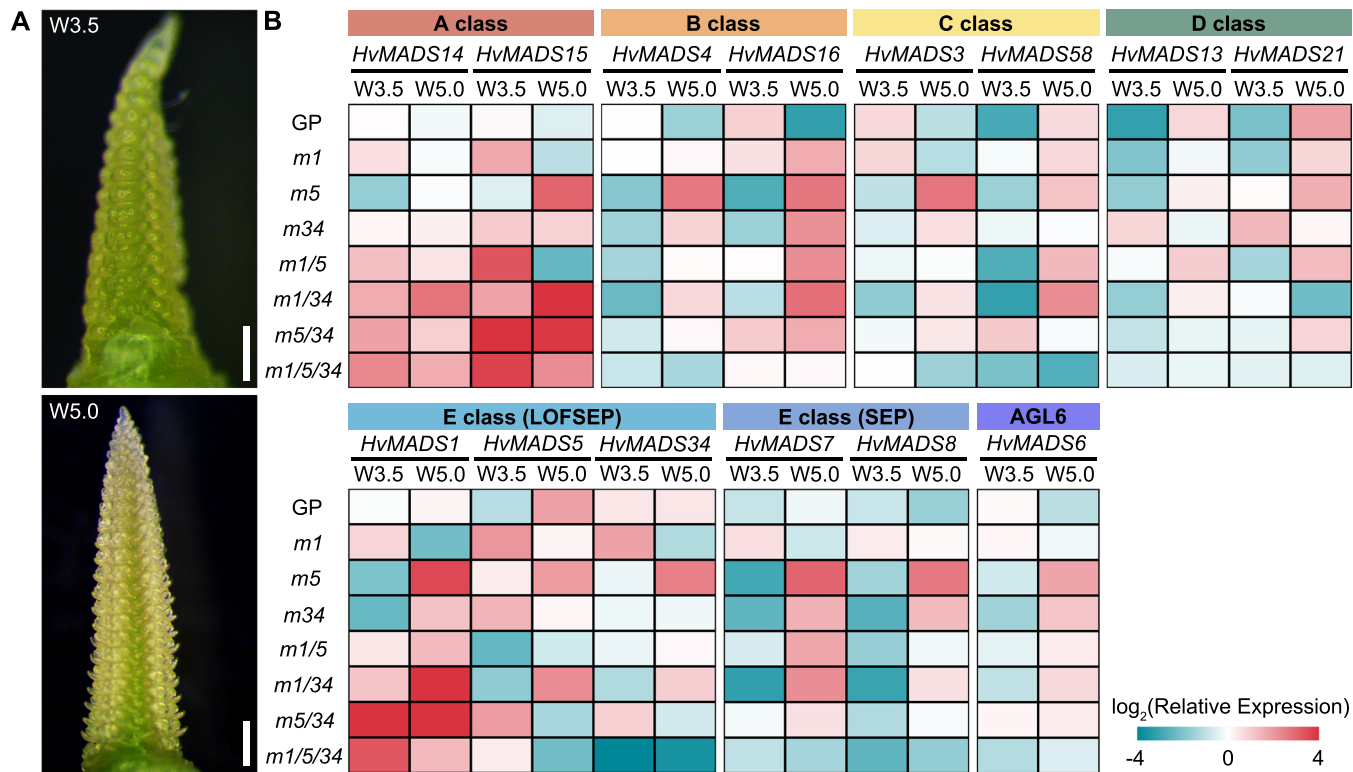

Supplement: Supplementary file 6 — Supplementary file6 (PDF 12051 kb) [file 42994_2024_182_MOESM6_ESM.pdf]
